# Comparative Effectiveness of Tunneling vs. Coronally Advanced Flap Techniques for Root Coverage: A 6–12-Month Randomized Clinical Trial

**DOI:** 10.3390/bioengineering12080824

**Published:** 2025-07-30

**Authors:** Luis Chauca-Bajaña, Pedro Samuel Vásquez González, María José Alban Guijarro, Carlos Andrés Guim Martínez, Byron Velásquez Ron, Patricio Proaño Yela, Alejandro Ismael Lorenzo-Pouso, Alba Pérez-Jardón, Andrea Ordoñez Balladares

**Affiliations:** 1 College Dentistry, University of Guayaquil, Guayas 090101, Ecuador; luis.chaucab@ug.edu.ec (L.C.-B.); patricio.proanoy@ug.edu.ec (P.P.Y.); andrea.ordonezb@ug.edu.ec (A.O.B.); 2 Periodontics and Implantology Oral Research, University of Guayaquil, Guayas 090101, Ecuador; dr.pedrovasquezgonzalez@gmail.com; 3 Specialist in Forensic Medicine, University of Guayaquil, Guayas 090101, Ecuador; maria.albang@ug.edu.ec; 4 School of Dentistry, Universidad Católica de Santiago de Guayaquil (UCSG), Guayas 090101, Ecuador; carlosandresguim@gmail.com; 5 Dental Prosthesis Department Research, College Dentistry, University of the Americas, UDLA, Av, Colon y 6 de Diciembre, Campus Colón, Quito 170102, Ecuador; 6 Oral Medicine, Oral Surgery and Implantology Unit (MedOralRes), Faculty of Medicine and Dentistry, University of Santiago de Compostela, 15782 Santiago de Compostela, Spain; alexlopo@hotmail.com (A.I.L.-P.); perlopjm@gmail.com (A.P.-J.); 7 College Dentistry, University Bolivariana del Ecuador, Durán 092406, Ecuador

**Keywords:** gingival recessions, surgical flaps, tissue conditioning dental, controlled clinical trials

## Abstract

Background: Gingival recession is a common condition involving apical displacement of the gingival margin, leading to root surface exposure and associated complications such as dentin hypersensitivity and root caries. Among the most effective treatment options are the tunneling technique (TUN) and the coronally advanced flap (CAF), both combined with connective tissue grafts (CTGs). This study aimed to evaluate and compare the clinical outcomes of TUN + CTG and CAF + CTG in terms of root coverage and keratinized tissue width (KTW) over a 6–12-month follow-up. Methods: A randomized, double-blind clinical trial was conducted following CONSORT guidelines (ClinicalTrials.gov ID: NCT06228534). Participants were randomly assigned to receive either TUN + CTG or CAF + CTG. Clinical parameters, including gingival recession depth (REC) and KTW, were assessed at baseline as well as 6 months and 12 months postoperatively using a calibrated periodontal probe. Statistical analysis was performed using descriptive statistics and linear mixed models to compare outcomes over time, with a significance level set at 5%. Results: Both techniques demonstrated significant clinical improvements. At 6 months, mean root coverage was 100% in CAF + CTG cases and 97% in TUN + CTG cases, while complete root coverage (REC = 0) was observed in 100% and 89% of cases, respectively. At 12 months, root coverage remained stable, at 99% in the CAF + CTG group and 97% in the TUN + CTG group. KTW increased in both groups, with higher values observed in the CAF + CTG group (3.53 mm vs. 3.11 mm in TUN + CTG at 12 months). No significant postoperative complications were reported. Conclusions: Both TUN + CTG and CAF + CTG are safe and effective techniques for treating RT1 and RT2 gingival recession, offering high percentages of root coverage and increased KTW. While CAF + CTG achieved slightly superior coverage and tissue gain, the TUN was associated with better aesthetic outcomes and faster recovery, making it a valuable alternative in clinical practice.

## 1. Introduction

Gingival recession or soft tissue retraction is characterized by the downward displacement of the gum line below the cement–enamel junction (ECU) of a tooth or the platform of a dental implant [1,2]. It causes loss of attachment and exposure of the root surface [3]. In the United States, it has been documented that approximately 58% of people over 30 years of age have gingival recession of ≥1 mm, and, on average, about 22.3% of teeth per individual are affected by this condition [4,5]. Yadav et al. (2023) [6], in a systematic review and meta-analysis, determined that more than two-thirds of the population is affected by gingival recession. At the Global Workshop on the Classification of Periodontal and Peri-implant Diseases, gingival recessions were described in the RT1, RT2, and RT3 categories [7]. RT1 is characterized by no loss of interproximal attachment, while RT2 includes interproximal attachment loss that is less than or equal to the buccal attachment loss. These criteria guided the selection of patients included in this study [7]. The consequences of gingival recession include dental hypersensitivity, root caries, and non-carious cervical lesions [8]. Currently, there are several surgical techniques to cover exposed roots [9,10], Various surgical techniques have been presented in order to maintain the health of the papilla, which is necessary to achieve complete root coverage or carry out regenerative therapy [11]. Connective tissue grafting is the gold standard in root-covering procedures to improve aesthetic appearance and protect exposed root surfaces [12]. In comparative studies, incorporating CTG across treatment groups has been considered a methodological strategy to ensure uniform regenerative potential and to isolate the effect of surgical flap design on clinical outcomes. Accordingly, both tunneling and coronally advanced flap techniques have often been combined with CTG to maximize root coverage and soft tissue integration. One of the surgical techniques studied is the tunneling technique, as it emerged as an effective approach for root coverage, with the potential to offer satisfactory aesthetic and functional results [13,14]. The tunneling technique is less invasive compared to other surgical procedures to cover roots, resulting in less discomfort, with faster recovery for the patient [15]. The tunneling technique with connective tissue grafting has been shown to be effective in the treatment of multiple gingival recessions [16,17,18]. Randomized clinical trials comparing the tunneling technique (TUN) with coronal flap advancement (CAF) have shown that both techniques are effective [19,20].

The aim of this randomized clinical trial was to compare the clinical efficacy of the tunneling technique (TUN) and the coronally advanced flap (CAF), both combined with connective tissue grafts, in the treatment of RT1 and RT2 gingival recessions, with follow-up evaluations at 6 and 12 months.

## 2. Materials and Methods

### 2.1. Study Design and Data Collection

This randomized, double-blind clinical trial was conducted in accordance with the CONSORT guidelines [21] and registered with ClinicalTrials.gov (ID: NCT06228534). Ethical approval was obtained from the Ethics Committee of the University of the Americas (CBE/UDLA17059/2024/04/08). All participants provided written informed consent prior to enrollment. Results of the 6–12-months follow-up of the randomized, double-blind clinical trial were used to evaluate the treatment of single and multiple gingival recessions using two surgical techniques: TUN and CAF, seeking to evaluate the efficacy in the coverage of gingival recession types RT1 and RT2 [22].

### 2.2. Participants

This study was conducted as a randomized clinical trial; however, due to its preliminary nature, no formal sample size calculation was performed prior to recruitment. A post hoc power analysis based on the observed data indicated that approximately 56 recession sites per group would be required to detect a clinically relevant difference of 0.64 mm in gingival recession depth, with 80% power and a 5% significance level. Therefore, while the current sample provides valuable initial insights into the comparative effectiveness of the techniques, the results should be interpreted with caution and considered as foundational evidence to inform the design of future larger-scale studies. Additionally, although the post hoc power analysis estimated 56 recession sites per group, only 37 sites were included due to recruitment challenges and strict eligibility criteria. This lower sample size may reduce the statistical power of the study, and results should be interpreted accordingly.

The inclusion criteria were

1. Age ≥ 18 years;
2. Non-smoker;
3. No systemic diseases;
4. No pregnancy;
5. No active periodontal disease;
6. Plaque and bleeding rate ≤ 9%;
7. Not taking medications that interfere with periodontal tissue health or healing;
8. Teeth without root canals;
9. No contraindication for periodontal surgery;
10. Presence of at least one recession defect type RT1 and/or RT2 in the upper or lower jaw that clearly showed the amelocementary boundary;
11. The treatments would be performed on the central incisors, lateral incisors, premolars, and molars, both upper and lower;
12. For the coronal advancement technique, multiple or single gingival recessions would be used.

Exclusion criteria were

1. Presence of active periodontal disease;
2. Being an active smoker;
3. Inadequate level of oral hygiene;
4. Furca involvement;
5. Uncorrected brushing trauma;
6. Severe dental malpositions;
7. History of previous root cover procedures.

**Figure 1:**
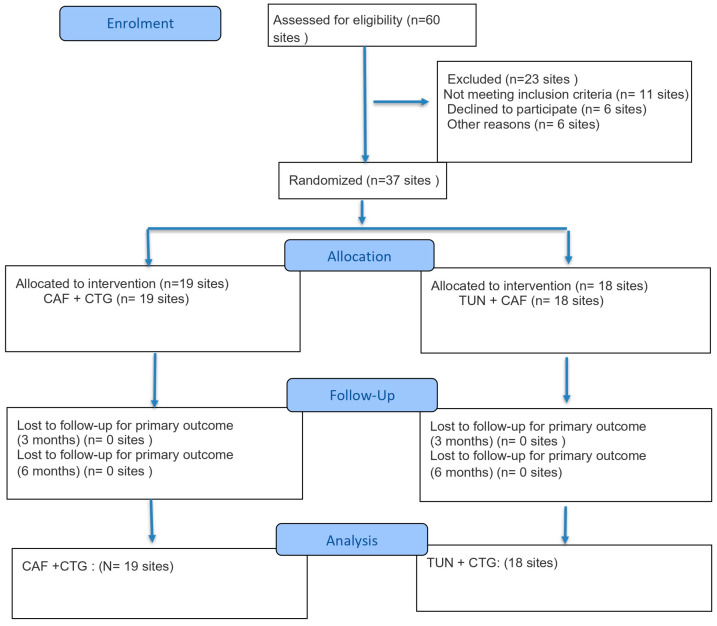
CONSORT flow diagram of the participants throughout this study.

Of the 60 patients assessed, 23 were excluded: 11 did not meet the inclusion criteria, 6 declined to participate, and 6 withdrew consent before randomization (Figure 1).

Figure 1 presents the CONSORT flow diagram of participant progress throughout this study.

### 2.3. Randomization

Randomization was performed using STATA software, and allocation was concealed using sealed opaque envelopes prepared by a blinded third party. The envelopes were opened by the operator only after local anesthesia was administered.

### 2.4. Surgical Interventions

The surgical interventions were performed by two trained specialists with extensive experience in periodontal plastic surgery (C.G. and P.V.). To ensure objectivity, two independent investigators blinded to the surgeries performed clinical evaluations, using a HU-FRIEDY North Carolina probe for the measurements. The test group was TUN + CTG, and the control group was CAF + CTG. Depth of recession as well as thickness and height of keratinized tissue were measured, as was the presence or absence of amelocementary line and/or step [23].

### 2.5. TUN + CTG (Test Group)

**Figure 2:**
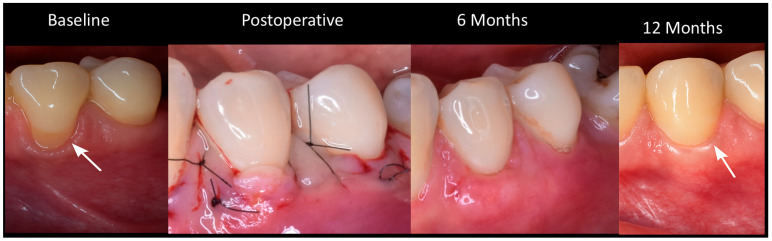
Clinical progression of root coverage with TUN: baseline to 12-month follow-up.

A tunnel is created by a partial-thickness incision in each recession area that will be addressed during the procedure. Special attention is paid to the manipulation of the tissue beyond the mucogingival junction in order to obtain a tension-free tunnel that facilitates the insertion of the graft. At the level of the interdental papillae, a careful incision is made, elevating the papillae gently without separating their tips. A palate graft that is 1 to 1.5 mm thick (measured using a calibrated periodontal probe) is removed, extending from the canine area to the first molar, making sure that it is long enough to cover the root of all the teeth involved. The graft is inserted into the tunnel using a specific suturing technique. Polydioxanone 6-0 thread (Vital Sutures, Fort Myers, FL, USA) was used. The first suture is passed through the most distal part of the recession, and the needle emerges in the most medial part of the recession. The second suture is placed on the opposite side; the needle also emerges in the same medial location of the recession. The graft, supported by both sutures (mesial and distal), slides smoothly into the tunnel, passing under the interdental papillae. Once the graft is in the desired position, both sutures are knotted to secure and stabilize the inserted graft, which exposes the graft in the area of recession [24,25] (Figure 2).

### 2.6. CAF + CTG (Control Group)

**Figure 3:**
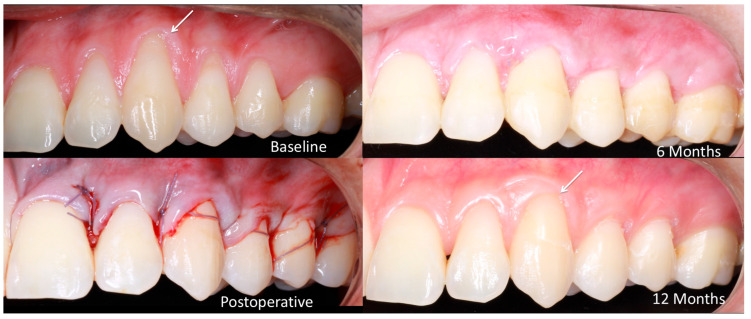
Clinical progression of root coverage with coronally advanced flap: baseline to 12-month follow-up.

After local anesthesia with articaine (3 m, ESPE; Schiller Park, IL, USA), according to the technique used, the preparation of the recipient area was carried out. In the flap technique after the measurements were carried out, it began with an incision at partial thickness by means of a 15c scalpel. Once the surgical papillae were ready, a flap was raised to full thickness until 3 mm of bone were visualized. With a new 15c scalpel, deep incisions were made, and then partial incisions were made parallel to the flap in order to free it from its muscle insertion. Once the flap was released, epithelialization of the anatomical papillae was initiated, and the root surfaces were finely scraped and smoothed using Gracey 11/12 or 1/2 curettes. The donor area was also anesthetized, and, after measurements, the trap-type graft introduced by Zucchelli began. Once the graft was taken, it was placed in saline solution, and the donor area was treated with a hemostatic sponge and X-stitches with 6-0 nylon thread. The graft was prepared using a new 15c scalpel, and the oral epithelium was removed. The graft was adapted in the recipient area using single stitches with polydioxanone 6-0 thread. Once the graft was stabilized, the flap was sutured with suspensory stitches in the same way with polydioxanone 6-0 suture thread. Finally, a force-breaking point was performed at the apical point of the recipient area [26,27,28] (Figure 3).

### 2.7. Postoperative Care

Patients were instructed not to brush their teeth in the treated area but to rinse their mouths with a chlorhexidine solution (0.12%) twice daily for 1 min. Fourteen days after surgical treatment, the sutures were removed. Plaque control in the surgically treated area was maintained by 0.05% chlorhexidine rinses for another 2 weeks. After this period, patients were again instructed in mechanical cleaning of the teeth of the treated region using an ultra-soft toothbrush. The stitches were removed between 7 and 10 days.

### 2.8. Post-Surgical Medication

A 60 mg ampoule of Ketorolac tromethamine (Dolgenal) was administered after the surgical procedure for pain control, with amoxicillin + clavulanic acid (1 g every 12 h) for 7 days as antibiotic treatment. For additional pain and inflammation management, 400 mg ibuprofen every 8 h for 3 days was indicated. Ketorolac tromethamine (Dolgenal Rapid), in 30 mg sublingual tablets, with a dose of one every 8 h for a period of 3 days, was recommended in the presence of severe pain. Patients were instructed to strictly comply with the pharmacological regimen and attend postoperative controls to evaluate evolution as possible complications.

### 2.9. Donor Area

**Figure 4:**
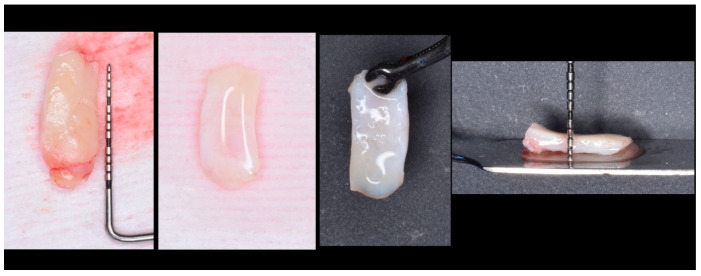
Harvesting, preparation, and standardization of the connective tissue graft.

Surgeries were initiated in all participants by extracting connective tissue from the mucosa of the palate using the de-epithelialized free gingival graft technique [29] (Figure 4).

### 2.10. Clinical Variables

Evaluations were performed at the end of surgery as well as 6 and 12 months after surgery using a HU-FRIEDY North Carolina periodontal probe. Clinical parameters such as the depth of the recession, from the gingival margin to the amelocementary boundary, and the width of the keratinized tissue were measured. Plaque and bleeding scores throughout the mouth (FMPS/FMBPS), type and location of gingival recession were also recorded. The mean percentage of root cover (mRC) and changes in the depth of the recession were also assessed.

### 2.11. Statistical Analysis

Statistical analyses were performed using SPSS v25. Descriptive statistics were used to summarize clinical parameters. Linear mixed models were applied to evaluate differences in outcomes (gingival recession depth and keratinized tissue width) across time points (baseline, 6 months, and 12 months) and treatment groups (CAF + CTG vs. TUN + CTG), accounting for repeated measures. A random intercept was included for each subject to account for within-subject correlations due to multiple measurements. Pearson’s correlation was used to assess the relationship between gingival recession depth and keratinized tissue width. A significance level of 5% (*p* < 0.05) and 95% confidence intervals were used for all comparisons. Additionally, for between-group comparisons, analysis of covariance (ANCOVA) was performed to adjust for baseline differences in clinical parameters such as gingival recession depth and keratinized tissue width. Baseline values were included as covariates to account for initial imbalances and improve the accuracy of treatment effect estimates.

## 3. Results

The sample consisted of 10 patients: 3 men (30%) and 7 women (70%), with a mean age of 38 years (±10.8; range: 24–65 years). Most treated teeth were located in the maxillary arch, with 72.9% corresponding to quadrants 1 and 2. At baseline, the average gingival recession (REC) was 0.6 mm (±1.1 mm), and the mean keratinized tissue width (KTW) was 2.8 mm (±1.2 mm), providing a reference point for evaluating treatment outcomes. A total of 37 gingival recession sites were treated and analyzed: 19 in the CAF + CTG group and 18 in the TUN + CTG group; demographic data are reported per patient, while clinical outcomes are provided per recession site (Table 1).

Initial comparisons between the two surgical groups revealed a statistically significant baseline difference in gingival recession depth (*p* = 0.042). The TUN + CTG group presented with more severe gingival recession (mean = 2.17 mm; SD = 1.29 mm) than the CAF + CTG group (mean = 1.45 mm; SD = 0.78 mm). Despite this initial imbalance, both groups demonstrated significant reductions in gingival recession depth at 6 months. It reduced in the TUN + CTG group to a mean of 0.11 mm (SD = 0.32 mm), while the CAF + CTG group achieved complete root coverage (mean REC = 0 mm). These results remained stable at 12 months. The TUN + CTG group maintained a mean gingival recession depth of 0.11 mm (SD = 0.32 mm), and the CAF + CTG group presented only a minimal recurrence, with a mean of 0.03 mm (SD = 0.11 mm). These findings support the sustained efficacy of both surgical techniques over time, though initial differences in severity should be considered when interpreting the comparative results (Table 2).

Keratinized tissue width (KTW) also showed significant and steady improvement during follow-up in both groups. Initially, KTW was lower in the TUN + CTG group (1.67 mm ± 0.97 mm) than in CAF + CTG (2 mm ± 0.88 mm). At 6 months, both groups showed a significant increase: TUN + CTG reached 2.67 mm (±0.84 mm), and CAF + CTG achieved 3.11 mm (±0.459 mm). At 12 months, this positive trend continued, reaching 3.11 mm (±1.18 mm) in TUN + CTG and 3.53 mm (±0.84 mm) in CAF + CTG (Table 3).

Statistical analysis at 6 months revealed significant improvements in both groups. In TUN + CTG, the REC decreased by an average of 2.05 mm (SD = 1.21 mm; CI: 1.45–2.66), while KTW increased by 1 mm (SD = 0.77 mm; CI: 0.62–1.38). In CAF + CTG, the REC reduced by an average of 1.45 mm (SD = 0.78 mm; CI: 1.07–1.82), while KTW increased by 1.11 mm (SD = 0.94 mm; CI: 0.65–1.56). Although both interventions showed statistically significant improvements within groups (*p* < 0.0001), intergroup comparisons adjusted for baseline values revealed no statistically significant differences between techniques (REC: *p* = 0.095; KTW: *p* = 0.772) (Table 4).

At 12 months, the results continued to be encouraging. In TUN + CTG, the REC decreased by an average of 2.06 mm (SD = 1.21 mm; CI: 1.45–2.66), and KTW increased by 1.44 mm (SD = 1.15 mm; CI: 0.87–2.02). On the other hand, in CAF + CTG, the REC reduced by an average of 1.42 mm (SD = 0.77 mm; CI: 1.05–1.79), and KTW increased 1.53 mm (SD = 0.9 mm; CI: 1.09–1.96). Both techniques demonstrated statistically significant within-group improvements over time (*p* < 0.0001). However, baseline-adjusted intergroup comparisons revealed no statistically significant differences between groups (REC: *p* = 0.082; KTW: *p* = 0.844) (Table 5).

Root cover showed highly positive results in both procedures. At 6 months, the TUN + CTG group achieved 97% coverage (SD = 0.09), while CAF + CTG achieved 100% complete coverage (SD = 0). At 12 months, TUN + CTG maintained coverage stable at 97% (SD = 0.01), and CAF + CTG showed a slight reduction, reaching 99% (SD = 0.06) (Table 6).

At 6 months, the CAF + CTG procedure achieved 100% total root coverage (REC = 0), while it was 89% in TUN + CTG. At 12 months, TUN + CTG maintained its coverage of 89%, showing stability over time, while it decreased slightly in CAF + CTG to 95% (Table 7).

The difference between the mean root coverage (Table 6) and the percentage of complete root coverage (Table 7) reflects the distinct outcome measures used. Table 6 reports the average percentage of root coverage achieved across all treated recession sites, including those with partial but substantial improvement. Table 7, on the other hand, reports only the proportion of sites that achieved complete root coverage (i.e., REC = 0 mm). This explains the slight difference in reported outcomes despite overall favorable results in both groups.

**Figure 5:**
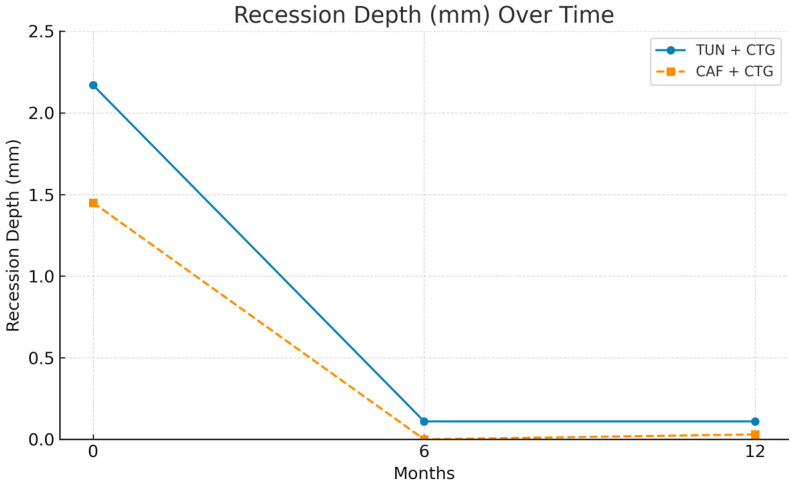
Multi-line graph for comparing surgical procedures between groups (REC).

Figure 5 shows that both surgical procedures, CAF and TUN, are effective in significantly reducing gingival recession (REC) from baseline to 6 months, achieving values close to zero. This reflects an almost complete coverage of the treated roots. Between 6 and 12 months of follow-up, both techniques maintained stable results, with CAF showing a slight advantage in the overall reduction in recession.

**Figure 6:**
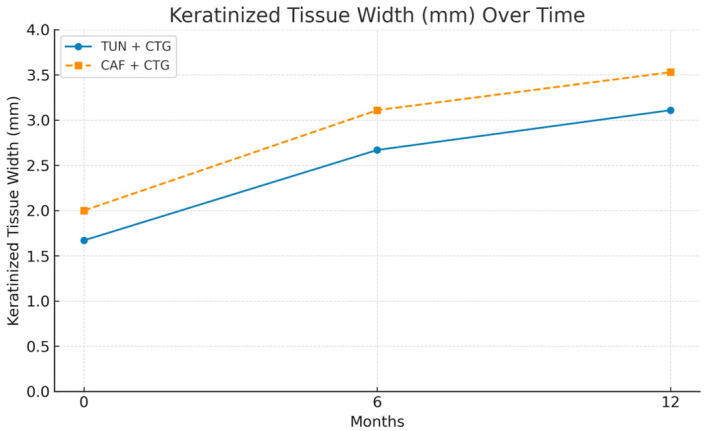
Multi-line graph for comparing surgical procedures between groups (KTW).

The increase in keratinized gingival width (KTW) showed notable differences. As can be seen in Figure 6, both CAF and TUN produced significant increases in keratinized gingiva width throughout the follow-up period. However, the CAF procedure produced a more consistent and marked increase, reaching higher values at both 6 and 12 months, compared to the TUN procedure.

**Figure 7:**
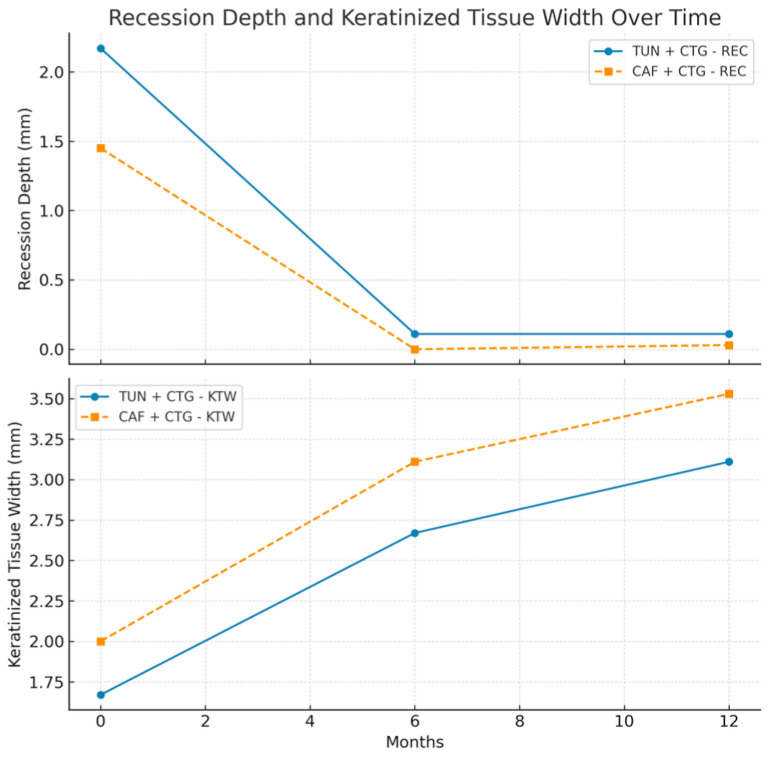
Multi-line graph for comparing surgical procedures between groups (REC + KTW).

The combined analysis of gingival recession indicators (RECs) and keratinized gingiva width (KTW) reinforces the effectiveness of both surgical procedures (Figure 7). The recession decreases drastically in the first 6 months and remains stable until 12 months. Keratinized gingiva shows a progressive and sustained increase throughout the evaluation period, which translates into clinical improvements that are functional and aesthetically relevant.

**Figure 8:**
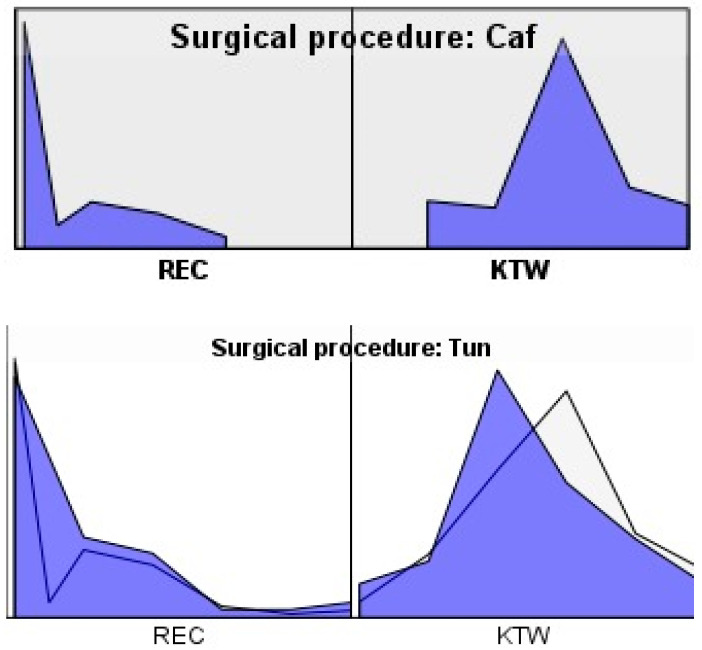
Graph for comparing subgroups between surgical procedures (REC + KTW).

Figure 8 illustrates the individual clinical response patterns observed for each recession site in both surgical groups (CAF and TUN), separated by clinical parameter: gingival recession depth (REC, left panel) and keratinized tissue width (KTW, right panel). Each line represents a single treated site. In the CAF group, most REC curves show an abrupt drop toward baseline values, indicating successful root coverage, while KTW values present marked variability, with some cases showing sharp increases. Conversely, in the TUN group, REC reductions were also consistent, though some variability was noted, and KTW values showed a more gradual and moderate increase across sites. The peak heights in the KTW plots represent the magnitude of tissue gain at individual sites, and the variability across curves highlights the heterogeneity in clinical response. The graphs were standardized in size and axis range to allow visual comparison. These patterns support the quantitative findings: both techniques were effective in reducing recession, but CAF showed a trend toward higher tissue keratinization, albeit with more inter-site variation.

## 4. Discussion

The TUN and CAF periodontal surgical techniques have both strengths and weaknesses. The CAF technique offers better visibility and access during dissection, as well as more effective graft stabilization by peristatic elevation [30]. However, TUN preserves the gingival papilla, which promotes faster healing and provides better blood supply to the graft, resulting in superior aesthetic results compared to CAF [14,15,16,17,18,19,20]. Both techniques, however, have limitations, which underlines the need to use specialized surgical material [31], such as connective tissue grafts (CTGs), which play a fundamental role in the reconstruction of the marginal gingiva [15,16,17,18,19,20,21,22,23,24,25,26,27,28,29,30,31,32].

This study aimed to evaluate the efficacy of TUN versus CAF, both combined with CTG, with controls performed at 6 and 12 months in relation to gingival recession (REC) and keratinized gingiva width (KTW). Initial results showed that the TUN + CTG group had a steeper recession (mean = 2.167 mm) compared to CAF + CTG (mean = 1.45 mm). However, at 6 months, both groups achieved significant reductions in the recession: TUN + CTG reached an average of 0.11 mm, while CAF + CTG achieved complete elimination (REC = 0 mm). At 12 months, the results remained stable, with CAF + CTG showing a slight residual recession (mean = 0.03 mm). These findings are consistent with several randomized controlled trials (RCTs) supporting the effectiveness of both TUN [13] and CAF [19] in recovering from gingival recession. Both techniques have been shown to provide satisfactory clinical results, favoring coverage of the exposed root and improving periodontal health in general. However, some authors present a different perspective by arguing that the combination of CAF + CTG results in a higher recession coverage rate [33]. This combination not only maximizes root coverage but also promotes better tissue integration, which is critical for the long-term success of periodontal regeneration. Recent studies have shown that the use of CTG together with CAF can be especially beneficial in cases of more severe recessions or in patients with unfavorable anatomical characteristics [34,35].

In our research, KTW showed significant improvement in both groups during follow-up. Initially, KTW was lower in the TUN + CTG group (1.67 mm) compared to CAF + CTG (2 mm). At 6 months, both groups showed an increase: TUN + CTG reached 2.67 mm, and CAF + CTG reached 3.11 mm. At 12 months, the results continued to show a positive trend, with TUN + CTG reaching 3.11 mm and CAF + CTG at 3.53 mm. However, a study conducted by González, which included 30 patients, investigated the TUN and CAF surgical techniques at 3 and 6 months. The results of that research indicate that both surgical interventions (TUN and CAF) showed similar efficacy in terms of root coverage, early healing and aesthetic results at 6 and 12 months. However, the TUN demonstrated a significantly greater increase in KTW, since in the study, CAF values of 2.6 ± 1.2 (N = 15) and TUN values of 2.3 ± 1.3 (N = 15) were reported, in addition to a shorter duration of surgery [2]. These results are opposite to those observed in other studies, where an increase in KTW in the CAF group compared to TUN [19] is evidenced. This discrepancy could be due to differences in the surgical TUN (full-thickness vs. split-thickness preparation) used and the placement of soft tissue used (cellular dermal matrix vs. CTG). The possibility that the greater increase in KTW observed in our study in TUN-treated areas was due to secondary keratinization of exposed CTG cannot be ruled out. However, it is unclear whether this difference will be sustained in the long term, as there is evidence that KTW can increase up to nine years after surgery when CAF is combined with a CTG [36].

Our study demonstrated that the TUN + CTG and CAF + CTG procedures are very effective in the treatment of gingival recession (REC). After 6 months, TUN + CTG achieved 97% coverage, while it was 100% in CAF + CTG. At 12 months, both techniques maintained positive results: the REC in TUN + CTG reduced by 2.06 mm, and the keratinized gingiva width increased by 1.44 mm, while the REC in CAF + CTG reduced by 1.42 mm, and the KTW raised by 1.53 mm. These findings coincide with those of Tavelli et al., who conclude that, for multiple (87.8%) and localized (82.7%) recessions, the CAF technique provides better root coverage compared to TUN, using either CTG or an acellular dermal matrix [30]. In addition, a systematic review and meta-analysis showed that the CAF technique presents superior results to TUN [37]. This statement is supported by previous studies that highlight the importance of KTW in improving aesthetics and periodontal health [19,30]. However, Mayta-Tovalino et al. (2023) point out that TUN and CAF offer similar results in primary and secondary phases [20]. These findings are consistent with several studies that indicate that both techniques are effective in achieving complete root coverage [10,38,39].

This research showed positive results in mean root coverage. At 6 months, it was 97% in TUN + CTG and 100% in CAF + CTG. Regarding total root coverage, it was 100% in CAF + CTG (REC = 0) and 89% in TUN + CTG. These results are supported by a study conducted by Santamaría et al., which reports a percentage of mean root coverage (CMR) of 87.2 ± 27.1% for the CAF + CTG group, compared to 77.4 ± 20.4% for the TT+CTG group (*p* = 0.02). In addition, in terms of total root coverage (TRC), the CAF + CTG group had the highest percentage, with 71.4%, in contrast to 28.6% in the TT+CTG group (*p* = 0.01) [40]. These findings reaffirm the results obtained.

Finally, it was determined that at 12 months, a stable coverage of 97% was maintained in TUN + CTG (SD = 0.01), while a slight reduction in CAF + CTG, reaching 99% (SD = 0.06) mean root coverage (CMR). Regarding total root coverage (CTR), it was stable in TUN + CTG over time, while it decreased slightly to 95% in CAF + CTG. Although no significant differences were observed in the results, this minimal variation in root coverages could be attributed to the nature of the graft used, with CTG being considered the gold standard for these procedures. Although CAF presents slightly lower values, the advantages of TUN include faster healing and superior aesthetics, making it the best alternative [13,14,15].

This study highlights the effectiveness of the TUN and CAF surgical techniques, both combined with connective tissue grafts (CTGs), in treating gingival recession. While CAF achieved slightly higher root coverage and greater tissue gain at 6 and 12 months, TUN stood out for preserving the gingival papilla, favoring faster healing and potentially superior esthetic outcomes. These findings suggest that technique selection should be based on individual patient needs, with TUN being preferable in esthetically demanding cases. This trial’s strengths include its randomized, double-blind design, standardized surgical protocols, and follow-up assessments at both 6 and 12 months.

The use of CTG in both treatment groups was a deliberate methodological choice aimed at optimizing clinical outcomes and allowing for a fair comparison between the two flap designs. CTG is considered the gold standard in root coverage procedures due to its proven benefits in increasing tissue thickness, achieving greater stability, and enhancing esthetic outcomes. By standardizing the grafting material across both techniques, the study design focused on isolating the effects attributable to the surgical approach (CAF vs. TUN). Nevertheless, we recognize that the use of CTG in both arms may have attenuated differences in performance between the techniques, highlighting its central role in achieving successful outcomes regardless of flap type.

However, this study has several limitations that should be considered when interpreting the results. First, the sample size was limited to 10 participants, which may restrict the generalizability of the findings. Although multiple sites were included per patient (mean of 3.7), intra-patient clustering may have introduced correlation in healing responses that was not fully accounted for, despite the use of mixed models. Second, the distribution of treated sites varied across quadrants, and anatomical differences—such as flap mobility, tissue biotype, and surgical access—may have influenced the clinical response, particularly in posterior regions. Third, only Miller Class I (RT1) and Class II (RT2) recessions were included, limiting applicability to more advanced defects. Additionally, all procedures were performed by a single experienced clinician, which, while controlling for technique variability, may not reflect outcomes in broader clinical settings. Finally, esthetic outcomes were not quantified using objective indices or patient-reported measures, limiting the strength of conclusions regarding visual improvements. Future trials should incorporate standardized esthetic assessments, such as the root coverage esthetic score (RES) or patient-reported outcome measures (PROMs), to validate these observations. Overall, both techniques proved highly effective for RT1 and RT2 recessions. While CAF may offer slightly better root coverage, TUN provides distinct advantages in soft tissue preservation and recovery. Future studies with larger samples, longer follow-up, and alternative graft materials are recommended to further validate these outcomes and optimize periodontal treatment strategies.

## 5. Conclusions

Tunneling techniques (TUNs) and advanced coronal flaps (CAFs), combined with connective tissue grafting (CTGs), are effective in treating gingival recession types RT1 and RT2. Both achieved high root coverage and increased keratinized tissue width, with stable results at 12 months. CAF + CTG showed a slight advantage in root coverage, while TUN + CTG stood out in aesthetics and fast recovery. Future studies with larger samples and longer follow-up could strengthen these findings.

## Figures and Tables

**Table 1:** Patient description and dental care.

Variable	Category/Range	n	%
Sex	Male	3	30.0%
	Female	7	70.0%
Age (Years)	Range	24–65	
	Mean ± SD	38 ± 10.8	
Treated Teeth (Quadrants)	Q1	13	35.1%
	Q2	14	37.8%
	Q3	7	18.9%
	Q4	3	8.1%
Baseline REC (mm)	Range	0–5	
	Mean ± SD	0.6 ± 1.1	
Baseline KTW (mm)	Range	0–5	
	Mean ± SD	2.8 ± 1.2	

**Table 2:** Description of recession in surgical procedures.

	Group	n	REC (mm)
Baseline	TUN + CTG	18	2.17 ± 1.29
	CAF + CTG	19	1.45 ± 0.78
6 Months	TUN + CTG	18	0.11 ± 0.32
	CAF + CTG	19	0.00 ± 0.00
12 Months	TUN + CTG	18	0.11 ± 0.32
	CAF + CTG	19	0.03 ± 0.11

**Table 3:** Description of keratinized gingiva in surgical procedures.

	Group	n	KTW (mm)
Baseline	TUN + CTG	18	1.67 ± 0.97
	CAF + CTG	19	2.00 ± 0.88
6 Months	TUN + CTG	18	2.67 ± 0.84
	CAF + CTG	19	3.11 ± 0.46
12 Months	TUN + CTG	18	3.11 ± 1.18
	CAF + CTG	19	3.53 ± 0.84

**Table 4:** Comparison between recession and keratinized gingiva (6 months).

Baseline—6 Months	REC				KTW		
	n	Average (mm)	SD (mm)	IC	Average (mm)	SD (mm)	IC
TUN + CTG	18	2.05	1.21	1.45–2.66	1	0.77	0.62–1.38
CAF + CTG	19	1.45	0.78	1.07–1.82	1.11	0.94	0.65–1.56
Intergroup *p*-Value	<0.095				<0.772		

Note: Intergroup comparisons were performed using ANCOVA adjusted for baseline values. No statistically significant differences were found (*p* > 0.05).

**Table 5:** Comparison between recession and keratinized gingiva (12 months).

Baseline—12 Months	n	REC (mm) (Average ± SD)	IC	KTW Average (mm)	SD (mm)	IC
TUN + CTG	18	2.06 ± 1.21	1.45–2.66	1.44 ± 1.15		0.87–2.02
CAF + CTG	19	1.42 ± 0.77	1.05–1.79	1.53 ± 0.90		1.09–1.96
Value *p* intergroup	<0.082			<0.844		

Note: Intergroup comparisons were performed using ANCOVA adjusted for baseline values. No statistically significant differences were found (*p* > 0.05).

**Table 6:** Mean root coverage at 6 and 12 months according to procedure.

	Group	n	Mean Root Coverage (%) ± SD
Baseline—6 months	TUN + CTG	18	97% ± 0.09
	CAF + CTG	19	100% ± 0.00
Baseline—12 months	TUN + CTG	18	97% ± 0.01
	CAF + CTG	19	99% ± 0.06

**Table 7:** Total root coverage at 6 and 12 months according to procedure.

	Group	n (REC = 0)	Total n	% Complete Root Coverage
Baseline—6 months	TUN + CTG	16	18	89%
	CAF + CTG	19	19	100%
Baseline—12 months	TUN + CTG	16	18	89%
	CAF + CTG	18	19	95%

Note: Fisher’s exact test revealed no statistically significant difference in complete root coverage at 12 months between groups (*p* = 0.609).

## Data Availability

All data are available upon request.

## References

[1] Imber J.C., Kasaj A. (2021). Treatment of Gingival Recession: When and How?. Int. Dent. J..

[2] González-Febles J., Romandini M., Laciar-Oudshoorn F., Noguerol F., Marruganti C., Bujaldón-Daza A., Zabalegui I., Sanz M. (2023). Tunnel vs. coronally advanced flap in combination with a connective tissue graft for the treatment of multiple gingival recessions: A multi-center randomized clinical trial. Clin. Oral Investig..

[3] Pini Prato G. (1999). Mucogingival deformities. Ann. Periodontol..

[4] Albandar J.M., Kingman A. (1999). Gingival recession, gingival bleeding, and dental calculus in adults 30 years of age and older in the United States, 1988–1994. J. Periodontol..

[5] Merijohn G.K. (2016). Management and prevention of gingival recession. Periodontol. 2000.

[6] Yadav V.S., Gumber B., Makker K., Gupta V., Tewari N., Khanduja P., Yadav R. (2023). Global prevalence of gingival recession: A systematic review and meta-analysis. Oral Dis..

[7] Jepsen S., Caton J.G., Albandar J.M., Bissada N.F., Bouchard P., Cortellini P., Demirel K., de Sanctis M., Ercoli C., Fan J. (2018). Periodontal manifestations of systemic diseases and developmental and acquired conditions: Consensus report of workgroup 3 of the 2017 World Workshop on the Classification of Periodontal and Peri-Implant Diseases and Conditions. J. Periodontol..

[8] Serino G., Wennström J.L., Lindhe J., Eneroth L. (1994). The prevalence and distribution of gingival recession in subjects with a high standard of oral hygiene. J. Clin. Periodontol..

[9] Cairo F., Barootchi S., Tavelli L., Barbato L., Wang H., Rasperini G., Graziani F., Tonetti M. (2020). Aesthetic-And patient-related outcomes following root coverage procedures: A systematic review and network meta-analysis. J. Clin. Periodontol..

[10] Zuhr O., Akakpo D., Eickholz P., Vach K., Hürzeler M.B., Petsos H., The Research Group for Oral Soft Tissue Biology & Wound Healing (2021). Tunnel technique with connective tissue graft versus coronally advanced flap with enamel matrix derivate for root coverage: 5-year results of an RCT using 3D digital measurement technology for volumetric comparison of soft tissue changes. J. Clin. Periodontol..

[11] Zuhr O., Akakpo D., Eickholz P., Vach K., Hürzeler M.B., Petsos H., The Research Group for Oral Soft Tissue Biology & Wound Healing (2020). Tunnel technique with connective tissue graft versus coronally advanced flap with enamel matrix derivate for root coverage: 2-year results of an RCT using 3D digital measuring for volumetric comparison of gingival dimensions. J. Clin. Periodontol..

[12] Rebele S.F., Zuhr O., Schneider D., Jung R.E., Hürzeler M.B. (2014). Tunnel technique with connective tissue graft versus coronally advanced flap with enamel matrix derivative for root coverage: A RCT using 3D digital measuring methods. Part II. Volumetric studies on healing dynamics and gingival dimensions. J. Clin. Periodontol..

[13] Zuhr O., Rebele S.F., Schneider D., Jung R.E., Hürzeler M.B. (2014). Tunnel technique with connective tissue graft versus coronally advanced flap with enamel matrix derivative for root coverage: A RCT using 3D digital measuring methods. Part I. Clinical and patient-centred outcomes. J. Clin. Periodontol..

[14] Cheng X., Tang R., Ge Z. (2023). Comparison of the efficacy and long-term stability of tunnel technique and coronally advanced flap in the treatment of gingival recession: A Meta-analysis. Hua Xi Kou Qiang Yi Xue Za Zhi.

[15] Aroca S., Molnár B., Windisch P., Gera I., Salvi G.E., Nikolidakis D., Sculean A. (2013). Treatment of multiple adjacent Miller class I and II gingival recessions with a Modified Coronally Advanced Tunnel (MCAT) technique and a collagen matrix or palatal connective tissue graft: A randomized, controlled clinical trial. J. Clin. Periodontol..

[16] Zabalegui I., Sicilia A., Cambra J., Gil J., Sanz M. (1999). Treatment of multiple adjacent gingival recessions with the tunnel subepithelial connective tissue graft: A clinical report. Int. J. Periodontics Restor. Dent..

[17] Zuhr O., Fickl S., Wachtel H., Bolz W., Hürzeler M.B. (2007). Covering of gingival recessions with a modified microsurgical tunnel technique: Case report. Int. J. Periodontics Restor. Dent..

[18] Aroca S., Keglevich T., Nikolidakis D., Gera I., Nagy K., Azzi R., Etienne D. (2010). Treatment of class III multiple gingival recessions: A randomized-clinical trial. J. Clin. Periodontol..

[19] Ozenci I., Ipci S.D., Cakar G., Yilmaz S. (2015). Tunnel technique versus coronally advanced flap with acellular dermal matrix graft in the treatment of multiple gingival recessions. J. Clin. Periodontol..

[20] Mayta-Tovalino F., Barboza J.J., Pasupuleti V., Hernandez A.V. (2023). Efficacy of Tunnel Technique (TUN) versus Coronally Advanced Flap (CAF) in the Management of Multiple Gingival Recession Defects: A Meta-Analysis. Int. J. Dent..

[21] Moher D., Hopewell S., Schulz K.F., Montori V., Gøtzsche P.C., Devereaux P.J., Elbourne D., Egger M., Altman D.G. (2012). CONSORT 2010 explanation and elaboration: Updated guidelines for reporting parallel group randomised trials. Int. J. Surg..

[22] Strauss F.J., Marruganti C., Romandini M., Cavalla F., Neira P., Jiménez F.J., Jung R.E., Sanz M., Aravena J.G. (2023). Epidemiology of mid-buccal gingival recessions according to the 2018 Classification System in South America: Results from two population-based studies. J. Clin. Periodontol..

[23] Cortellini P., Bissada N.F. (2018). Mucogingival conditions in the natural dentition: Narrative review, case definitions, and diagnostic considerations. J. Clin. Periodontol..

[24] Cairo F. (2017). Periodontal plastic surgery of gingival recessions at single and multiple teeth. Periodontol. 2000.

[25] Mandil O., Sabri H., Manouchehri N., Mostafa D., Wang H.L. (2023). Root coverage with apical tunnel approach using propolis as a root conditioning agent: A case report with 2-year follow-up and review of the literature. Clin. Exp. Dent. Res..

[26] de Sanctis M., Zucchelli G. (2007). Coronally advanced flap: A modified surgical approach for isolated recession-type defects: Three-year results. J. Clin. Periodontol..

[27] Kuis D., Sciran I., Lajnert V., Snjaric D., Prpic J., Pezelj-Ribaric S., Bosnjak A. (2013). Coronally advanced flap alone or with connective tissue graft in the treatment of single gingival recession defects: A long-term randomized clinical trial. J. Periodontol..

[28] Zucchelli G., Marzadori M., Mounssif I., Mazzotti C., Stefanini M. (2014). Coronally advanced flap + connective tissue graft techniques for the treatment of deep gingival recession in the lower incisors. A controlled randomized clinical trial. J. Clin. Periodontol..

[29] Zucchelli G., Mele M., Stefanini M., Mazzotti C., Marzadori M., Montebugnoli L., De Sanctis M. (2010). Patient morbidity and root coverage outcome after subepithelial connective tissue and de-epithelialized grafts: A comparative randomized-controlled clinical trial. J. Clin. Periodontol..

[30] Tavelli L., Barootchi S., Nguyen T.V.N., Tattan M., Ravidà A., Wang H.L. (2018). Efficacy of tunnel technique in the treatment of localized and multiple gingi-val recessions: A systematic review and meta-analysis. J. Periodontol..

[31] Cheng G., Fu E., Tu Y., Shen E., Chiu H., Huang R., Yuh D., Chiang C. (2015). Root coverage by coronally advanced flap with connective tissue graft and/or enamel matrix derivative: A meta-analysis. J. Periodontal Res..

[32] Cieślik-Wegemund M., Wierucka-Młynarczyk B., Tanasiewicz M., Gilowski Ł. (2016). Tunnel Technique with Collagen Matrix Compared with Connective Tissue Graft for Treatment of Periodontal Recession: A Randomized Clinical Trial. J. Periodontol..

[33] Sanz-Martín I., Regidor E., Navarro J., Sanz-Sánchez I., Sanz M., Ortiz-Vigón A. (2020). Factors associated with the presence of peri-implant buccal soft tissue dehiscences: A case-control study. J. Periodontol..

[34] Tavelli L., Barootchi S., Avila-Ortiz G., Urban I.A., Giannobile W.V., Wang H.L. (2021). Peri-implant soft tissue phenotype modification and its impact on peri-implant health: A systematic review and network meta-analysis. J. Periodontol..

[35] Tavelli L., Majzoub J., Kauffmann F., Rodriguez M.V., Mancini L., Chan H.L., Kripfgans O.D., Giannobile W.V., Wang H.L., Barootchi S. (2023). Coronally advanced flap versus tunnel technique for the treatment of peri-implant soft tissue dehiscences with the connective tissue graft: A randomized, controlled clinical trial. J. Clin. Periodontol..

[36] Rasperini G., Acunzo R., Pellegrini G., Pagni G., Tonetti M., Prato G.P.P., Cortellini P. (2018). Predictor factors for long-term outcomes stability of coronally advanced flap with or without connective tissue graft in the treatment of single maxillary gingival recessions: 9 years results of a randomized controlled clinical trial. J. Clin. Periodontol..

[37] Chauca-Bajaña L., Pérez-Jardón A., Silva F.F.V.E., Conde-Amboage M., Velásquez-Ron B., Padín-Iruegas E., Pérez-Sayáns M. (2024). Root Coverage Techniques: Coronally Advancement Flap vs. Tunnel Technique: A Systematic Review and Meta-Analysis. Dent. J..

[38] Azaripour A., Kissinger M., Farina V.S.L., Van Noorden C.J., Gerhold-Ay A., Willershausen B., Cortellini P. (2016). Root coverage with connective tissue graft associated with coronally advanced flap or tunnel technique: A randomized, double-blind, mono-centre clinical trial. J. Clin. Periodontol..

[39] Tavelli L., Barootchi S., Di Gianfilippo R., Modarressi M., Cairo F., Rasperini G., Wang H. (2019). Acellular dermal matrix and coronally advanced flap or tunnel technique in the treatment of multiple adjacent gingival recessions. A 12-year follow-up from a randomized clinical trial. J. Clin. Periodontol..

[40] Santamaria M.P., Neves F.L.d.S., Silveira C.A., Mathias I.F., Fernandes-Dias S.B., Jardini M.A.N., Tatakis D.N. (2017). Connective tissue graft and tunnel or trapezoidal flap for the treatment of single maxillary gingival recessions: A randomized clinical trial. J. Clin. Periodontol..

